# Group Size Predicts Social but Not Nonsocial Cognition in Lemurs

**DOI:** 10.1371/journal.pone.0066359

**Published:** 2013-06-26

**Authors:** Evan L. MacLean, Aaron A. Sandel, Joel Bray, Ricki E. Oldenkamp, Rachna B. Reddy, Brian A. Hare

**Affiliations:** 1 Department of Evolutionary Anthropology, Duke University, Durham, North Carolina, United States of America; 2 Department of Anthropology, University of Michigan, Ann Arbor, Michigan, United States of America; 3 Department of Biology, Northern Michigan University, Marquette, Michigan, United States of America; 4 Center for Cognitive Neuroscience, Duke University, Durham, North Carolina, United States of America; CNR, Italy

## Abstract

The social intelligence hypothesis suggests that living in large social networks was the primary selective pressure for the evolution of complex cognition in primates. This hypothesis is supported by comparative studies demonstrating a positive relationship between social group size and relative brain size across primates. However, the relationship between brain size and cognition remains equivocal. Moreover, there have been no experimental studies directly testing the association between group size and cognition across primates. We tested the social intelligence hypothesis by comparing 6 primate species (total N = 96) characterized by different group sizes on two cognitive tasks. Here, we show that a species’ typical social group size predicts performance on cognitive measures of social cognition, but not a nonsocial measure of inhibitory control. We also show that a species’ mean brain size (in absolute or relative terms) does not predict performance on either task in these species. These data provide evidence for a relationship between group size and social cognition in primates, and reveal the potential for cognitive evolution without concomitant changes in brain size. Furthermore our results underscore the need for more empirical studies of animal cognition, which have the power to reveal species differences in cognition not detectable by proxy variables, such as brain size.

## Introduction

Primates are characterized by large brains relative to their body sizes [1]. The social brain hypothesis has provided an explanatory framework for this phenomenon, suggesting that the cognitive demands of large social groups have favored greater degrees of encephalization in primate species, including humans [2]–[6]. However, the cognitive consequences of large brains remain unknown, and largely untested [7]–[9]. Therefore, direct measures of cognition are required in order to study the evolution of cognitive processes [8], [10].

The social intelligence hypothesis has been proposed as a major explanatory framework for primate cognitive evolution, and states that group living has favored the evolution of cognitive skills for competing with conspecifics for access to food and mates while maintaining and monitoring social relationships in large, stable, social groups [11]–[18]. One proposed factor determining social complexity is the size of the groups in which individuals of any species typically live in nature [5]. Therefore a central prediction of the social intelligence hypothesis is that social group size should correlate positively with cognitive skills across species. This hypothesis has received tentative support by way of paired comparisons of closely related species living in social groups of differing sizes [19], [20]. However, it is currently unknown whether there is a robust linear relationship between group size and cognitive skills observed in primates, or any other taxa.

A second important question concerns which cognitive traits are expected to respond to evolutionary changes in social systems. The domain-specific hypothesis predicts that larger social group sizes should lead to selection for cognitive skills that are specific to social living [21]. For example, animals living in large groups would benefit from cognitive skills allowing them to monitor and infer relationships between individuals, to cooperate with conspecifics, and to outcompete others for access to critical resources such as food and mates. However, other cognitive skills unrelated to group living should remain relatively unaffected. For example, the demands on cognitive skills related to navigation and foraging are less likely to change as a result of evolutionary fluctuation in social group sizes. In essence, this hypothesis predicts mosaic cognitive evolution, in which different cognitive skills change relatively independently of one another. In contrast, the domain-general hypothesis argues for the existence of a general intelligence factor (g) and asserts that cognitive traits for reasoning about the social and nonsocial world are not independent of one another [22]. Accordingly, this hypothesis predicts that any cognitive changes favored by group living should be similar for both social and nonsocial cognition.

In the current experiments we compared the cognitive skills of six lemur species characterized by different species-typical group sizes in a social and nonsocial cognitive task. We explored the relationship between performance on these tasks and a species’ typical social group size, as well as the relationship between performance and absolute or relative brain size. By incorporating a social and nonsocial cognitive test we aimed to measure whether any associations between group size and performance were specific to social cognition [21] or whether these relationships generalized to nonsocial cognitive skills as well [16], [22].

The social-cognitive task measured a subject’s ability to exploit positional information indicative of a competitor’s visual perspective. Proponents of the social intelligence hypothesis have emphasized the utility of these skills for group living species [18], [23]. For example, these skills would be particularly useful when low-ranking individuals attempt to acquire food resources, or mating opportunities in secrecy from other group members. The non-social task measured a subject’s capacity for inhibitory control, a cognitive process underlying decision making in diverse cognitive domains, which has been linked to health, academic, and economic success in humans [24], [25]. Recently, performance on this task has also been shown to correlate with song repertoire size in sparrows, a factor predictive of reproductive success [26].

From an evolutionary perspective, lemurs provide an ideal clade for comparison on these tasks due to their genetic similarity but varying degrees of sociality [27]–[29]. Further, unlike anthropoid primates, social group sizes are not correlated with brain size among lemurs permitting a natural experiment in which the variables of group size and brain size are independent of one another in the comparative sample [30]. Our sample included six lemur species with varying typical social group sizes. *Lemur catta* are characterized by a multi-male, multi-female social system and reside in the largest groups of any lemur species (mean group size: 15.6) [31]. *Eulemur fulvus* and *Eulemur macaco* have the next largest social groups in our sample, and live in multi-male, multi-female groups, which are smaller than those of *Lemur catta* (mean group sizes – *Eulemur fulvus*: 8.5; *Eulemur macaco*: 9.9) [32], [33]. *Propithecus coquereli* and *Varecia variegata* live in groups ranging from adult pairs, to small multi-male, multi-female groups (mean group sizes – *Propithecus coquereli*: 6.1; *Varecia variegata*: 5.4) [34], [35]. *Eulemur mongoz* are characterized by the smallest social groups in our sample (mean group size: 3), and typically reside in pair-bonded family units [36].

### Ethics Statement

**Figure 1:**
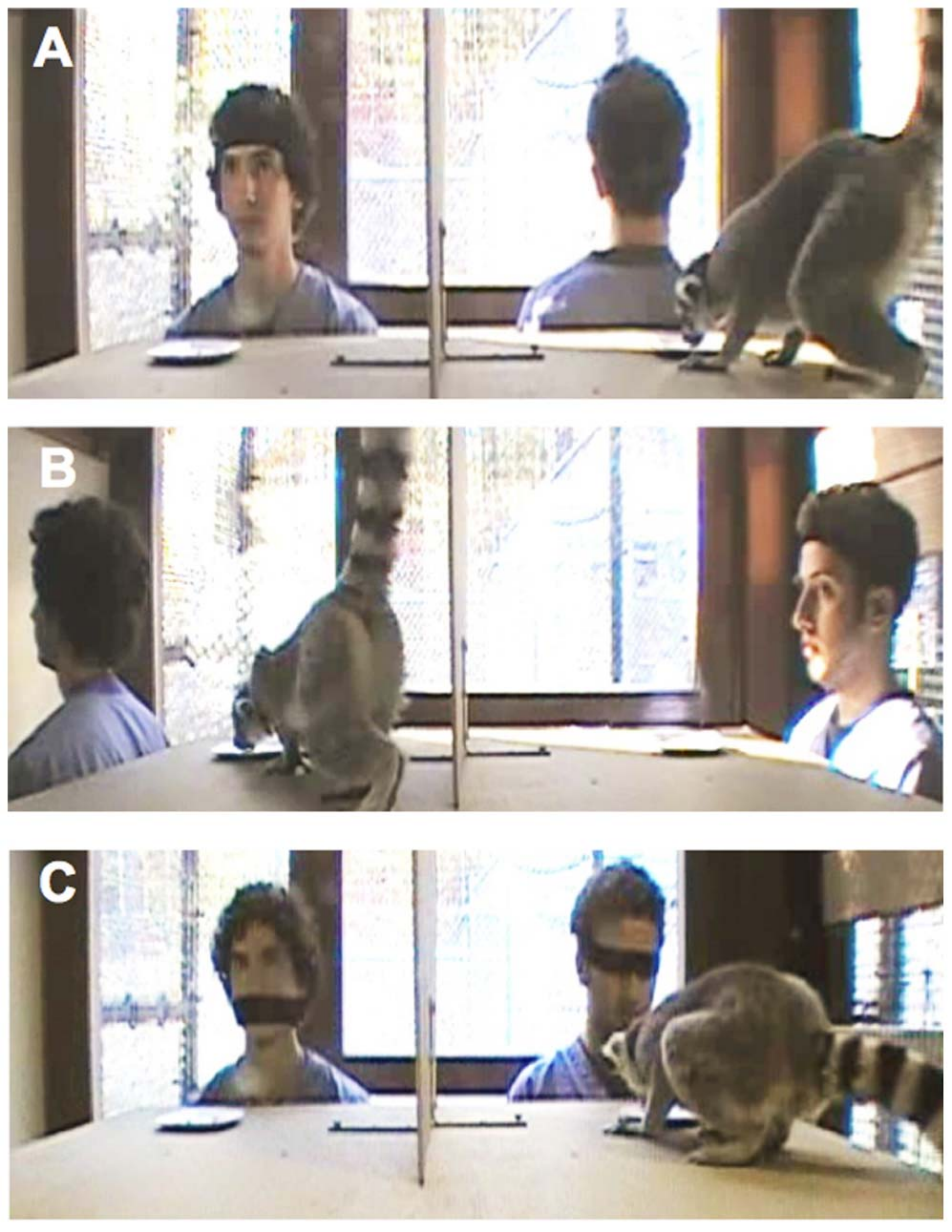
The test trials for Experiment 1. Subjects were given the opportunity to pilfer food from one of two human competitors. In each condition, one of the competitors could see the food and the subject approaching, while the other could not because A) his back was turned B) he was oriented away from the food in profile, or C) a headband covered his eyes.

All Experimental Procedures were approved by the Duke University Institutional Animal Care and Use Committee (Protocol #: A199-11-08). The individuals in the photograph in Figure 1 and Movie S1 have given written informed consent, as outlined in the PLOS consent form, to publication of their photograph and video appearance.

## Experiment 1

### Method

#### Subjects

We tested 10 brown lemurs (*Eulemur fulvus*; 6 female, 4 male), 10 black lemurs (*Eulemur macaco*; 4 female, 6 male), 10 mongoose lemurs (*Eulemur mongoz*; 4 female, 6 male), 10 ring-tailed lemurs (*Lemur catta*; 4 female, 6 male), 10 Coquerel’s sifakas (*Propithecus coquereli*; 4 female, 6 male), and 10 ruffed lemurs (*Varecia variegata*; 4 female, 6 male). Details regarding the sample are shown in Table S1 in File S1. All subjects were housed at the Duke Lemur Center in Durham, NC, USA. Subjects were socially housed (as pairs or small to large groups) in indoor-outdoor enclosures with various substrates provided for environmental enrichment (e.g. perches, nest boxes, climbing structures and ropes). All subjects were familiar with humans through daily routines including feeding and cleaning of the animal enclosures. Details regarding the facility and housing conditions can be found online at: http://lemur.duke.edu/research/facilities/. Subjects were fed a diet of primate biscuits, fresh fruit and vegetables, and water was available *ad libitum*. The majority of *Eulemur macaco*, *Eulemur mongoz*, *Lemur catta*, and *Varecia variegata* subjects had been tested previously in a similar study approximately 1 year before the current experiments [37] (Table S1 in File S1). All subjects were tested in their home enclosure, physically separated from all other group members for the duration of the session. Food was temporarily removed during the experimental session, but water was available ad libitum.

#### Apparatus

Lemurs were tested on an elevated platform (Figure S1 in File S1; 79×122×122 cm, H×L×W). A vertical panel (61×61 cm, H×L) was mounted on this platform to separate the choice locations used in the test (Figure S1 in File S1). Food was presented on small rectangular plates that could be taken on and off the platform over the course of trials (Movie S1).

#### Procedure

The procedure was based on previous studies of visual perspective taking conducted with lemurs, monkeys, and apes [37]–[39]. The experiment consisted of four phases: (1) an introductory phase, (2) pre-test, (3) test, and (4) a post-test. In the introductory phase the subject was first attracted (with food) to a centering block at one end of the platform that was located equidistantly from the two choice locations at the other end of the platform (Figure S1 in File S1). Two experimenters placed a piece of food (grape pieces for all species except *Propithecus coquereli* who received nuts or leaves due to dietary differences) in the choice locations opposite from the subject (Figure S1 in File S1). The experimenters then faced away from the subject until the lemur had eaten the food at both choice locations. This trial served both to familiarize the lemur with the locations where food could be positioned and to assure that the subject had experience feeding from both locations. All subsequent trials began by first attracting the subject to the centering block with a food reward.

Pre-test trials served to introduce the subject to the competitive nature of the task. In the pre-test, one experimenter placed two baited food trays on opposite sides of the panel separating the choice locations and then sat on a stool (97 cm height) directly behind one of the trays with her face level with the tray. If a subject approached the food in front of the experimenter (contested food), the experimenter quickly removed this tray (taking it off the table and out of the subject’s view) and the subject was allowed 1 minute to feed from the uncontested food tray. If the subject approached the uncontested food tray she was allowed to feed freely and was given 1 minute to approach the contested food tray. If the subject approached the contested food tray during this period, the experimenter quickly removed the tray. In all trials a subject was scored as having approached a food tray if her head or hand came within 5 cm of the food tray. Four pre-test trials were conducted; the choice location that was defended, and the identity of the experimenter defending the food (E1 or E2) were counterbalanced within subjects. Lemurs were required to choose the uncontested food for the last three pretest trials in order to advance to the test. If a subject did not meet this criterion, the session was interrupted and a new session was administered after a short break (∼5 minutes). No more than 4 sessions were conducted on any day to assure that subjects would be motivated for food throughout test trials. If the subject did not participate within 15 minutes (i.e. would not approach a choice location) the session was interrupted and these subjects were eligible to be tested at a later date. Thirteen subjects began the pre-test but did not advance to test trials due to failure to meet the pretest criterion or participation requirements in the time they were available for study.

Test trials were identical to pre-test trials except that two experimenters were present, one behind each choice. In each trial, one of these experimenters could see the food and the subject (contested choice) while the other could not (uncontested choice). In the ‘front-back’ condition one experimenter faced the food while the other experimenter turned her back (Figure 1A). In the ‘profile’ condition both experimenters were oriented to the lemur in profile but one of the experimenters could see the food because it was in front of her face while the other experimenter could not see the food because it was positioned behind her head (Figure 1B). In the ‘eyes-mouth’ condition, both experimenters faced forward and one experimenter could not see the food because she wore a headband covering her eyes, while the other experimenter could see the food because she wore the headband covering her mouth (Figure 1C). In all test trials, if the lemur approached the uncontested food, she was allowed to feed. If the lemur approached the contested choice, the experimenter guarding this choice quickly removed the food preventing the lemur from feeding (Movie S1). On ½ of the trials, subjects were given 1 minute to self-correct (i.e. to approach the uncontested food) following an incorrect choice, in order to encourage continued participation in the task. On all trials if a lemur did not approach either food item within 2 minutes the trial was interrupted, the subject was re-centered, and a new trial was administered. If a subject did not approach 2 times for any particular trial, this trial was scored as ‘no choice’ and the next trial was administered. Subjects were required to make a choice on at least 8/12 trials in order to be included in the analyses.

We conducted 4 trials of each condition (administered in a block). All subjects received the ‘front-back’ condition first. The order of the ‘profile’ and ‘eyes-mouth’ conditions was counterbalanced between subjects. The location of the contested choice and the identity of the experimenter defending the food were counterbalanced within subjects and condition so that choices based on side biases or reputational effects would lead to chance performance. Following the test trials we conducted 4 post-test trials that were identical to the pre-test trials administered at the beginning of the session. These trials verified that subjects were capable of performing the basic discrimination (choosing the uncontested food when only one experimenter was present) and were motivated to obtain food through the end of the procedure. As in the pre-test, the location that was defended and identity of the experimenter defending the food (E1 or E2) were counterbalanced within subjects.

#### Analysis

Because data showed deviation from normality in some cases (see File S1), we used nonparametric statistics for all non-phylogenetic analyses. For all tests with a directional prediction, we used a directional hypothesis testing framework following the conventions (δ = 0.01, Υ = 0.04) recommended by Rice & Gaines [40]. Accordingly, the null hypothesis was rejected when the 1-tailed p value was ≤.04 in the predicted direction, or ≥.99 in the unanticipated direction. For all directional predictions (denoted below) we report the one-tailed p value. We compared performance to chance expectation (50%) using one-sample Wilcoxon tests to evaluate the directional hypothesis that subjects would target the uncontested food. We compared species using a Kruskal Wallis test with Dunn-Bonferroni post hoc comparisons. To evaluate the relationship between social group size, brain size, and performance we used phylogenetic generalized least squares to control for the non-independence of species level data. We incorporated the parameter λ to scale the internal branches of the phylogeny using the maximum likelihood estimate for the model [41]. All phylogenetic analyses were performed using a consensus tree from the 10K trees project [42]. We tested the directional predictions that group size and brain size would be positively associated with performance on the cognitive task. Scores from each of the three test conditions were averaged within each subject as an overall score for the task. A coder blind to the experimental condition scored 20% of trials from video to assess inter-observer reliability, which was excellent [43] (Kappa = 0.96).

### Results

#### Overall performance

**Figure 2:**
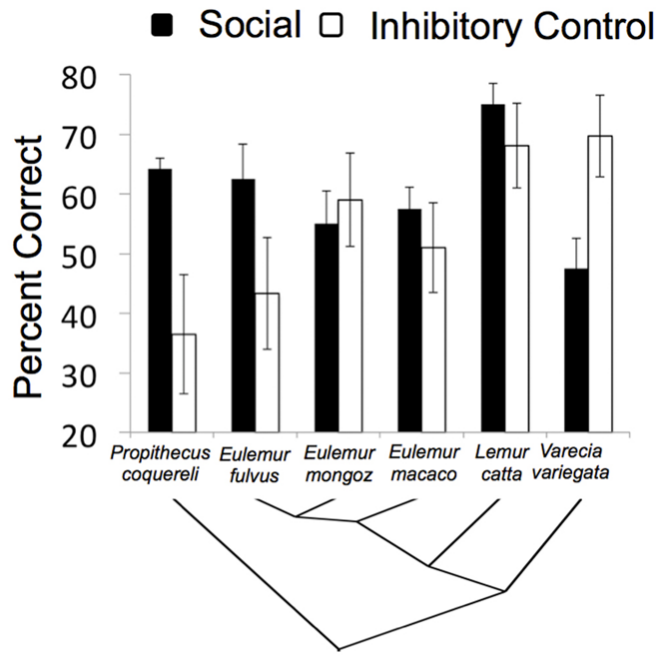
Species’ performance on the social cognition and inhibitory control tasks. The tree structure at the bottom of the figure represents the phylogenetic relationships between the species. Error bars reflect the standard error of the mean.

Overall, lemurs preferentially targeted the uncontested food when their competitor could not see because his back was turned (67.5±3.2% correct, W = 816, N = 60, p<.001), or because he was oriented in profile away from the food (62.1±3.5% correct, W = 814, N = 60, p<.001). Subjects did not differ from chance expectation when the only cue to a competitor’s awareness was whether a headband covered his eyes or mouth (51.2±2.9% correct, W = 321, N = 60, p = .33). In the post-test, lemurs were again successful at avoiding the food guarded by a single competitor who faced them (82±3% correct, W = 1166.5, N = 60, p<.001). The results for individual species are shown in Table 1 and Figure 2. A direct comparison of the 6 species’ overall scores indicated significant species differences (H = 16.88, df = 5, p<.01) and Dunn-Bonferroni post hoc comparisons revealed that *Lemur catta* scored higher than *Varecia variegata* (adjusted p = .002), but that no other pairs differed significantly (all adjusted p’s >.05).

**Table 1:** Scores (percent correct) from the social cognition task.

	Overall		Front vs. Back		Profile		Eyes vs. Mouth	
Species	Score ± SE	p value	Score ± SE	p value	Score ± SE	p value	Score ± SE	p value
*Lemur catta*	75±4	<.01	90±4	<.01	75±5	<.01	60±8	.14
*Eulemur mongoz*	55±6	.20	60±8	.12	58±8	.19	48±7	.61
*Varecia variegata*	48±5	.68	50±9	.5	38±9	.91	55±7	.26
*Eulemur macaco*	58±4	.05	70±7	.02	63±8	.08	40±7	.90
*Propithecus coquereli*	64±2	<.01	63±6	.05	68±9	.05	63±6	.04
*Eulemur fulvus*	63±6	.04	73±8	.02	73±9	.03	43±7	.83

Performance in each condition was compared to chance expectation (50%) using one-sample Wilcoxon tests to evaluate the hypothesis that subjects would attempt to steal the food that their competitor could not see.

#### Comparison of species within conditions

Species differed in the number of pretests required to meet the criterion (H = 15.88, df = 5, p = .007), and post hoc comparisons revealed that *Varecia variegata* required significantly more pretests than *Lemur catta* (adjusted p = .004), but that no other pairs differed (all adjusted p’s >.05). There were significant species differences in the front-back condition (H = 15.71, df = 5, p<0.01) and post-hoc tests revealed that *Lemur catta* outperformed *Varecia variegata* (adjusted p = .007) with no significant differences between any other pairs (all adjusted p’s >.05). In the profile condition, there were again significant species differences (H_ = _11.32, df = 5, p = .05) but post hoc tests revealed no significant pairwise differences (all adjusted p’s >.05) Lastly, there were no significant species differences in the eyes-mouth condition (H = 7.45, df = 5, p = .19), the only cue for which lemurs were not above chance as a group.

#### Relationship with social group size

**Figure 3:**
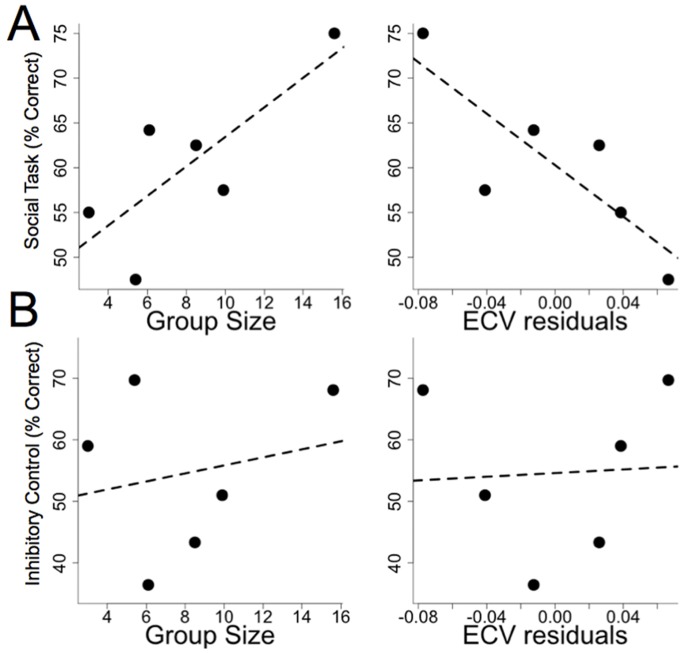
Performance in the cognitive tasks as a function of social group size and residual endocranial volume (ECV), a measure of relative brain size. We tested the hypotheses that social group size and relative brain size would predict species performance. A) As predicted by the social intelligence hypothesis, species characterized by larger social groups performed better in the social cognition task. Relative brain size did not explain species’ performance, and the slope of the relationship was negative. B) Group size did not predict performance on the non-social inhibitory control task. As in the social task, relative brain size was not a predictor of performance on the inhibitory control task.

The social intelligence hypothesis predicts that species living in large social groups should possess enhanced social-cognitive abilities relative to species that live in smaller social groups. To test this hypothesis we used phylogenetic generalized least squares (PGLS) [41], [44] to assess the relationship between a species’ typical group size [45] and its performance in the social-cognitive task while statistically controlling for the non-independence of species level data. Overall, group size was positively correlated with performance on the social-cognitive task (Figure 3; β = 1.65, t_4_ = 2.47, p = .03). This relationship was strongest for the front versus back cue (β = 2.79, t_4_ = 4.23, p<0.01) and positive, but not statistically significant for the profile cue (β = 1.8, t_4_ = 1.76, p = .08). There was no relationship between group size and species’ scores in the eyes versus mouth condition, the only cue for which lemurs were not above chance as a group (β = −0.2, t_4_ = −0.28, p = .60). Averaging data across the two conditions in which lemurs were above chance (front vs. back and profile cues) yielded similar results to the analysis averaging data across all three conditions (β = 2.3, t_4_ = 2.53, p = .03).

Because *Lemur catta* form the largest social groups of any species in our sample, and also performed best in this task, we conducted the above analyses without this species to evaluate the relationship between group size and performance without this species in the analysis. In this smaller comparative sample group size was significantly related to performance in the front vs. back condition (β = 1.90, t_3_ = 2.79, p = .03), but not in the profile (β = 2.2, t_3_ = .85, p = .23) or eyes vs. mouth condition (β = −1.12, t_3_ = −1.90, p = .92). Lastly, we repeated the above analyses using only data from subjects that participated in both Experiment 1 and Experiment 2. The results from these analyses were consistent with the analyses using all subjects, and are reported in File S1.

#### Relationship with brain size

To assess whether the cognitive differences we observed were related to brain size, we conducted the same analyses using two measures of brain size as the predictor variables. Relative brain size was calculated using the residuals from a PGLS model of log brain mass predicted by log body mass for the species in our sample. Absolute brain size was measured as log brain mass without controlling for body mass. The values for brain and body masses were species’ means from a published dataset [46]. Relative brain size did not predict performance in the social-cognitive task (Figure 3; β = −143.8, t_4_ = −2.92, p = .98), and the regression coefficient for brain size was negative. Similar analyses for each social cue revealed that brain size did not predict performance in any condition (front-back: β = −215.7, t_4_ = −3.23, p = .98; profile: β = −182.4, t_4_ = −2.06, p = .95; eyes-mouth: β = 8.21, t_3_ = .13, p = .45). The same analyses using absolute brain size as the predictor variable yielded similar results. Absolute brain size did not predict overall performance (β = −16.67, t_4_ = −.65, p = .72) and again the regression coefficient was negative. Similarly absolute brain size did not predict performance in any of the individual conditions (front-back: β = −37.30, t_4_ = −1.08, p = .83; profile: β = −33.24, t_4_ = 0. −.93, p = .80; eyes-mouth: β = −11.60, t_4_ = −0.59, p = .71).

### Discussion

As a group, lemurs successfully targeted the food that a competitor could not see in two of three experimental conditions. Specifically, lemurs were above chance when the discrimination involved the competitors’ body and head orientation (front-back and profile conditions) but did not distinguish between the attentive and inattentive competitors when the only difference was whether a headband covered their eyes or mouth. Thus, while lemurs are sensitive to some social cues relevant to others’ perception, they may not exploit more subtle cues involving only the eyes, as do some anthropoid species [38], [47].

We also observed significant species differences on this task. Although species varied in the number of sessions required to meet the pre-test criterion, all subjects met this criterion before the test, permitting a valid comparison of performance across species during the test. As predicted by the social intelligence hypothesis, the size of a species’ social group was positively correlated with social cognitive skills relevant to assessing a competitor’s awareness. These findings replicate and extend those of Sandel et al. (2011) who reported that ring-tailed lemurs, which have the largest social groups of any lemur species, performed better than three other closely related species in a similar social-cognitive task. The current results build on these findings using three novel social cues and a larger sample of species, and directly assess the relationship between group size and performance on the social-cognitive task. In contrast to the correlation with group size, brain size did not predict variance in cognitive skills, and the slope of the relationship between brain size and performance was negative in most cases. Thus, the results of Experiment 1 support the social intelligence hypothesis and demonstrate the potential for cognitive differences between species not detectable by proxy variables such as brain size.

One important point to consider is that four of the six species in our sample participated in a similar study approximately one year beforehand [37]. Although these subjects were faced with an entirely novel set of cues in this study it is possible that their previous experiences provided an advantage in the tests reported here. Importantly however, this fact is unlikely to account for the relationship between group size and performance that we observed. First, the two species without any prior experience performed as well or better than three of the four species that did have relevant previous experience. Thus, previous experience was not a prerequisite for success in this task. Secondly, both the best and worst performing species in our sample were among the species tested by Sandel et al. (2011) suggesting that previous experience did not uniformly improve or hinder performance in the current task. Consequently, while previous experience is always an important consideration, it does not account for main findings in Experiment 1.

## Experiment 2

In Experiment 1 we observed species differences on a social cognition task that correlated with group size, corroborating the predictions of the social intelligence hypothesis. However, it is unclear whether these cognitive differences are specific to social cognition, or whether they reflect more general cognitive differences between species. To address this question in Experiment 2, we compared the same species from Experiment 1 on a nonsocial task measuring inhibitory control. If the cognitive differences we observed in Experiment 1 reflect domain-general cognitive differences between species [48], [49] we predicted that we should observe the same relationship between group size and performance in a second nonsocial cognitive task. However, if the species differences from Experiment 1 are specific to social cognition [21], we predicted that performance on a nonsocial cognitive task would not bear the same relationship with a species’ typical group size.

### Method

#### Subjects

We tested 10 brown lemurs (*Eulemur fulvus*; 5 female, 5 male), 10 black lemurs (*Eulemur macaco*; 4 female, 6 male), 10 mongoose lemurs (*Eulemur mongoz*; 4 female, 6 male), 11 ring-tailed lemurs (*Lemur catta*; 6 female, 5 male), 10 Coquerel’s sifaka (*Propithecus coquereli*; 4 female, 6 male), and 11 ruffed lemurs (*Varecia variegata*; 5 female, 6 male). Testing and housing conditions were identical to those described above and details regarding the sample are shown in Table S1 in File S1.

#### Apparatus

**Figure 4:**
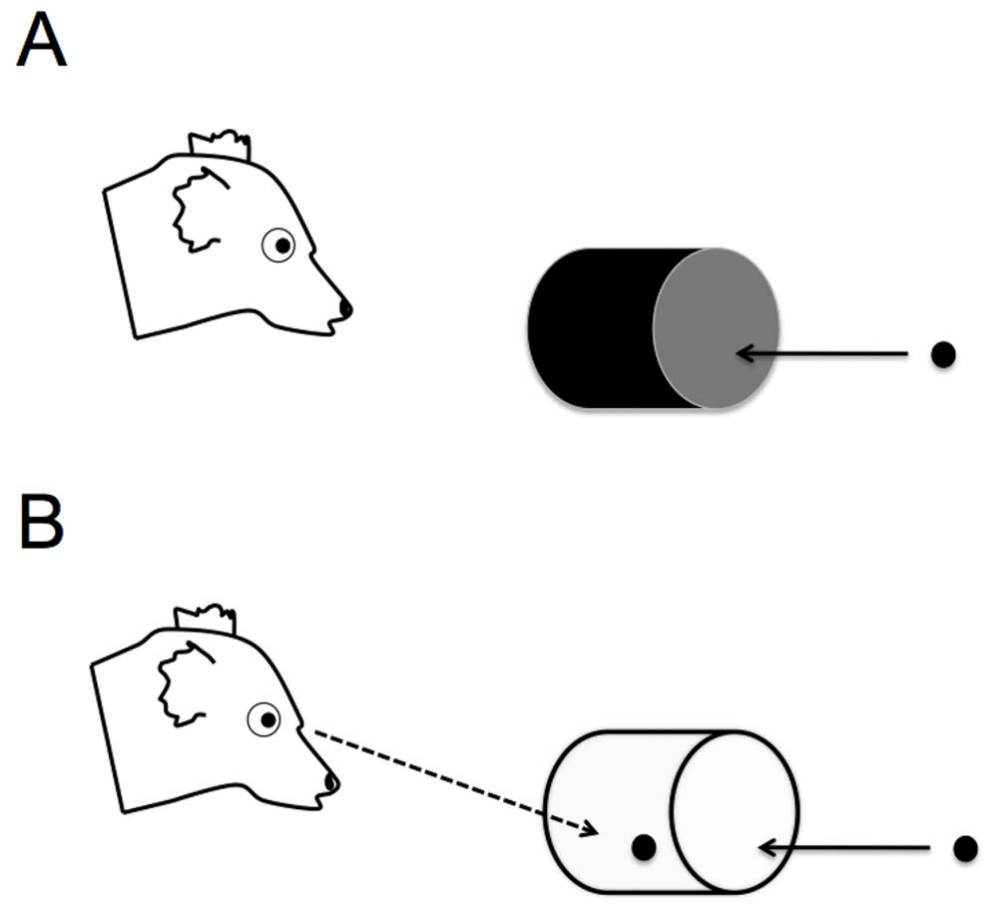
The apparatus for Experiment 2. In warm-up trials (A) the cylinder was opaque, preventing the subject from seeing the food while approaching. In test trials (B) the cylinder was transparent inducing the temptation to approach the visible food directly.

The apparatus consisted of two plastic cylinders (length-15 cm, diameter-10 cm), one opaque and one transparent, each attached to a different wooden block (23×28 cm; Figure 4). The wooden block served to keep the cylinder stationary throughout the trial so that subjects were required to adjust their approach to the apparatus, rather than manipulating the position of the apparatus itself when retrieving food.

#### Procedure

The procedure was based on previous studies of detour reaching in human infants and nonhuman primates [50]–[53] and consisted of familiarization and test trials. The familiarization trials served to habituate subjects to the apparatus and give them experience retrieving a piece of food from within the cylinder. Lemurs were first attracted (with a food reward) to a stationing block 1 m in front of the apparatus. Once the subject oriented towards the experimenter, the experimenter showed the subject a piece of food and placed it inside the opaque cylinder. The subject was then allowed to approach and retrieve this item. On every trial the experimenter coded whether the subject’s first attempt to retrieve the item was through the front of the apparatus (incorrect) or from the side (correct–successful detour). Subjects were permitted to retrieve the food reward on all trials regardless of the accuracy of their first attempt. If the subject did not approach within 30 seconds, the cylinder was re-baited and the trial was repeated. Subjects were required to retrieve the food reward (on their first attempt) by detouring to the side of the cylinder in 4 of 5 consecutive trials before advancing to the test. Once this criterion was met subjects advanced immediately to test trials. The warm-up was limited to 10 trials and if subjects did not meet the criterion during these 10 trials the session was interrupted. These subjects could be retested at a later time (4 subjects). The side from which the apparatus was baited was consistent within subjects but counterbalanced between subjects.

Test trials were identical to the warm-up trials except that the transparent cylinder was used (Figure 4). Thus lemurs were required to inhibit the desire to approach the visible food directly, in favor of a detour to the side of the apparatus where the food could be retrieved. We conducted 10 trials with all subjects. As in warm-up trials, the subject was first attracted (with a food reward) to the stationing block 1 m in front of the apparatus. Once the subject oriented towards the experimenter, the food reward was placed inside the transparent cylinder and the subject was allowed to approach and retrieve this item. The experimenter coded whether subjects’ first attempt to retrieve the item was through the front (incorrect) or the side (correct) of the apparatus (Movie S2). Again, subjects were allowed to retrieve the food item on all trials regardless of the accuracy of their first attempt. If the subject did not approach the testing area within 15 minutes or exhibited signs of stress (e.g. pacing, excessive scent marking, refusal to eat food), the session was interrupted (18 sessions) and the subject was eligible to be retested at a later time. Six subjects began the procedure but never advanced to test trials. If a subject had completed 8 or more test trials at the time the session was interrupted, the data were included for analysis (6 subjects; all scores were converted to percentages to account for differences in the total number of trials).

#### Analysis

The analyses were identical to Experiment 1. A second individual coded 100% of responses from video to assess inter-observer reliability (15 trials could not be coded due to an error in the video recordings). Inter-observer reliability was excellent [43] (Kappa = 0.91).

### Results

Overall, lemurs performed the correct response by reaching around the barrier on 55±4% of trials. Performance improved significantly between the first and second half of trials (1^st^ half: mean = 45±4, 2^nd^ half: mean = 65±4; W = 1236, N = 62, p<.01). There were no significant species differences in overall test scores (H = 10.73, df = 5, p = .06). However, species differed significantly in the first half of test trials (H = 13.15, df = 5, p = .02) and post hoc tests revealed that *Varecia variegata* scored higher than *Propithecus coquereli* (adjusted p = .05) but that no other pairs differed significantly (all adjusted p’s >.05). There were no significant differences in the second half of test trials (H = 6.78, df = 5, p = .24).

As in Experiment 1 we used PGLS to examine whether performance on the nonsocial task varied as a function of group size, as well as relative or absolute brain size. Unlike the social task, group size did not predict overall performance on the non-social inhibitory control task (Figure 3; β = 0.65, t_4_ = 0.44, p = .34). Because species varied most in the first half of trials, we repeated this analyses using only these data. The results of this analysis were similar to the analysis using all trials, and there was no relationship between group size and performance (β = 0.006, t_4_ = 0.005, p = .50).

Similarly to the social task, neither relative brain size (Figure 3; β = 14.86, t_4_ = .12, p = .46) nor absolute brain size (β = −37.99, t_4_ = −1.28, p = .87) predicted species’ performance on the inhibitory control task. Analysis of only the first half of trials yielded similar results (relative brain size – β = −15.5, t_4_ = -.14, p = .55; absolute brain size – β = −21.69, t_4_ = -.62, p = .72). Lastly, for subjects that participated in both Experiment 1 and Experiment 2, we assessed the correlation between tasks. Scores from these tasks were not correlated (r_s_ = -.07, N = 26, p = .74).

### Discussion

As in Experiment 1, we observed significant species differences in performance. However, in contrast to Experiment 1, performance on the nonsocial inhibitory control task was not correlated with a species’ typical group size. This finding suggests that the species differences we observed in Experiment 1 do not reflect generalized differences in the cognition of these species, but rather domain-specific differences that very likely relate to the natural histories of these species. However, it is important to note that while this task was intended as a measure of nonsocial problem solving, it included some potentially social elements in that subjects’ witnessed the experimenter bait the apparatus. Nonetheless, the lack of correlation between individuals’ performance in the two tasks provides further support for the possibility of domain-specific cognitive differences in our sample. Again we found no association between either measure of brain size and performance on the cognitive task.

## General Discussion

Across two experiments we observed significant differences in the cognitive skills of 6 lemur species. Our main finding was that a species’ typical group size predicted its performance in the social cognition task, but not the nonsocial inhibitory control task. Moreover, brain size (measured absolutely or relatively) did not predict performance in either task illustrating the importance of using actual cognitive measures for the study of cognitive evolution. To our knowledge these data provide the first demonstration of a link between group size and experimental measures of social cognitive skills in animals.

In Experiment 1 lemurs were required to pilfer food from one of two human competitors. In each case, one of the experimenters was able to see the lemur, while the other was not, because his vision was blocked or directed away from the subject. Overall lemurs were successful with cues regarding the positional orientation of their competitor (front/back of body, or head turned in profile), but were not above chance when the only information regarding which competitor could see them was whether a headband covered the eyes or mouth. These findings build on previous studies of social cognition in lemurs, and suggest that these primates are sensitive to a number of behavioral indications of others’ perception [37], [54]–[57]. Further, our data suggest that skills in this domain are related to a species’ typical group size, implicating a possible evolutionary relationship between sociality and cognitive skills for outcompeting others for access to contestable resources. These skills appear to be flexibly deployed, as lemurs were able to detect social cues from human experimenters. The extent to which these capacities mirror lemurs’ skills for reasoning about conspecifics remains an important topic for future research.

In Experiment 2 lemurs were required to resist the prepotent response to reach directly for visible food in favor of a detour response. Although this task measured skills for solving a physical problem (i.e. manually reaching around the barrier), it is possible that subjects used social information from the baiting procedure to guide their responses. Nonetheless, unlike Experiment 1, variability in this task was not predicted by a species’ group size suggesting that the findings from Experiment 1 do not reflect highly generalized species differences in problem-solving skills. These results are consistent with an analysis by Reader et al. in which scores from a variety of nonsocial cognitive tasks were not correlated with group size [22]. One implication of these results is that species differences in cognition are likely to be domain-specific and functionally related to the environments in which species have evolved [21], [58]–[60]. Interestingly the best performing species on the inhibitory control task was the highly frugivorous *Varecia variegata*, whose small social groups occasionally exhibit fission-fusion dynamics [34]. Fission-fusion dynamics have been proposed as another potential proxy of social complexity [61] and recently, in a comparative study of 7 primate species, Amici et al. [62] reported an association between fission-fusion dynamics and cognitive skills for inhibitory control. Although only one species in our sample is characterized by possible fission-fusion dynamics, it is notable that this species performed exceptionally well in the inhibitory control task.

Our finding that performance in the social cognitive task correlated with group size corroborates the predictions of the social intelligence hypothesis that species that live in larger social groups should require more flexible cognitive skills for competing with conspecifics. Previously this hypothesis has been supported primarily through comparative studies documenting a relationship between sociality and some indirect proxy for cognitive abilities. Most notably, several authors have shown that group size, or grooming network size is a good predictor of relative neocortex size in anthropoid primates [6], [63], [64]. However, the cognitive consequences of having a relatively large brain are largely unknown [65], but see [66].

The danger of conflating brain size with cognitive abilities [8] is made especially salient through these studies. Although we observed significant species differences in both experiments, this variation did not relate to variation in brain size. Thus, these data contribute to a growing number of studies in which smaller-brained species have outperformed larger-brained species in various cognitive tasks [20], [67]–[70]. Of course this is not to deny a possible functional relationship between brain size and cognition as these factors were almost certainly related during the course of human evolution [71]. However, the accumulation of findings violating a strict 1-to-1 relationship between these traits warrants great caution when making inferences about cognition based solely on brain size. Nonetheless, it is possible that larger comparative samples, or more refined data on brain component volumes and functional region connectivity, will more accurately predict variance in animal cognition [7], [72]–[74].

A final important question relevant to this study concerns the relative contributions of ontogeny and phylogeny in shaping species-typical cognitive abilities. The subjects in this study were housed at the Duke Lemur Center (DLC), and when possible, were maintained in species-typical groups. Consequently, it is likely that individuals of species characterized by large group sizes in the wild also resided in (and in some cases matured in) large social groups at the DLC. Because the individuals in this study have long and varied housing histories, it is impossible to directly assess the role of the ontogenetic environment in this case. However, studies exploring the environmental influences on neuroanatomy [75] and cognition [76] highlight the importance of the ontogenetic environment in shaping the adult neural and cognitive phenotype. Because our subjects tended to reside in species-typical social groups, we believe our data come from a sample that accurately represents species-typical cognitive traits.

Lastly, it is unknown whether the patterns reported here are similar to those for anthropoid primates (monkeys and apes). Unlike anthropoid primates, group sizes and relative brain sizes are not correlated in lemurs suggesting that the selective forces driving brain evolution may differ between these primate clades [30]. One possibility is that the smaller social groups of lemur species do not impose the same informational demands as the larger social networks characteristic of anthropoid primates [77]. This effect could be driven by the lower number of individual associations that any particular group member must keep track of, or by the nature of social interactions themselves. For example, lemur agonistic encounters are typically dyadic, and do not involve the agonistic alliances commonly observed in anthropoids [78], [79]. Nonetheless, these data suggest the possibility of cognitive evolution in the absence of corresponding changes in brain size.

## Supporting Information

File S1 **Supplemental analyses, Table S1, and Figure S1.**
(DOCX)

Movie S1 **The procedure for Experiment 1.**
(MOV)

Movie S2 **The procedure for Experiment 2.**
(M4V)
