# Supplementary material for: Group Size Predicts Social but Not Nonsocial Cognition in Lemurs
Source: PLoS One. 2013 Jun 26;8(6):e66359. doi: 10.1371/journal.pone.0066359 (PMC3694165; doi:10.1371/journal.pone.0066359)

**File S1**

**Supplemental Analyses**

*Normality tests.* We conducted Shapiro-Wilk tests for each of the cognitive variables included in the primary analyses. Because the data deviated from normality in the majority of cases we used nonparametric statistics for all non-phylogenetic analyses (social task – overall score: W = 0.96, N = 60, p = .07; social task – front vs. back condition: W = .88, N = 60, p < .01; social task – eyes vs. mouth condition: W = .90, N = 60, p < .01; social task – profile condition: W = .90, N = 60, p < .01; inhibitory control task – overall score: W = .96, N = 62, p = .02; inhibitory control task – first half trials: W = .92, N = 62, p < .01; inhibitory control task – second half trials: W = .89, N = 62, p < .01).

*Subset analyses.*  We conducted the same analyses used with all subjects including only the subset of individuals that participated in both tasks. Due to the low N in several species, these results should be interpreted cautiously (N’s: *Lemur catta* = 1, *Eulemur mongoz* = 7, *Eulemur macaco* = 4, *Eulemur fulvus* = 7, *Varecia variegata* = 5, *Propithecus coquereli* = 2). Results from these analyses mirrored those for the larger sample of lemurs. Specifically group size covaried positively with overall performance in the social task (β = 2.05, t_4_ = 3.0, p = .02), as well as with performance in the front vs. back (β =2.90, t_4_ = 2.58, p = .03) and profile conditions (β = 1.48, t_4_ = 2.86, p = .02). There was no relationship between group size and scores on the eyes vs. mouth condition, the only cue for which lemurs were not above chance as a group (β = 1.57, t_4_ = 1.21, p = .15). Neither absolute, or relative brain size predicted overall scores on the social task (absolute brain size: β = -24.08 , t_4_ = -.83, p = .77; relative brain size: β = -162.36 , t_4_ = -2.70, p = .97). Scores on the nonsocial inhibitory control task were not related to group size (β = 3.52, t_4_ = 1.93, p = .06) or either measure of brain size (absolute brain size: β = -47.58, t_4_ = -0.79, p = .76; relative brain size: β = -239.36, t_4_ = -1.54, p = .90)

Table S1. Subject information. Asterisks indicate participation in an experiment. Sandel et al. 2011 tested a subset of our subjects in a similar paradigm approximately 1 year beforehand.

| **Species** | **Subject** | **Sex** | **Age (y)** | **Social Task** | **Sandel et al. 2011** | **Inhibitory control task** |
| --- | --- | --- | --- | --- | --- | --- |
| *Eulemur fulvus* | Alphonse | M | 29 |  |  | * |
|  | Fiery | M | 27 |  |  | * |
|  | Francoise | F | 26 | * |  | * |
|  | Frigga | F | 27 | * |  | * |
|  | Geraldine | F | 22 | * |  | * |
|  | Giscard | M | 23 | * |  | * |
|  | Jules | M | 23 |  |  | * |
|  | Kish | M | 24 | * |  | * |
|  | Martine | F | 24 | * |  | * |
|  | Matthan | M | 25 | * |  |  |
|  | Pascal | M | 18 | * |  |  |
|  | Taphenes | F | 23 | * |  |  |
|  | Wizard | F | 16 | * |  | * |
| *Eulemur macaco* | Barrymore | M | 20 |  |  | * |
|  | Belushi | M | 2 | * | * |  |
|  | Deucalion | M | 22 | * | * |  |
|  | Foster | F | 12 |  | * | * |
|  | Harlow | F | 16 |  |  | * |
|  | Harmonia | F | 27 | * |  |  |
|  | Hesperus | M | 25 |  |  | * |
|  | Hopkins | M | 15 | * | * |  |
|  | L'amour | F | 22 | * | * | * |
|  | Latona | F | 27 | * |  |  |
|  | Margret | F | 1 | * |  |  |
|  | Olivier | M | 17 |  | * | * |
|  | Quinn | M | 10 | * |  | * |
|  | Redford | M | 19 | * | * | * |
|  | Tarantino | M | 12 | * | * | * |
|  | Teucer | M | 20 |  | * | * |
| *Eulemur mongoz* | Concepcion | F | 26 | * |  | * |
|  | Eduardo | M | 15 |  |  | * |
|  | Felipe | M | 15 | * | * |  |
|  | Flor | F | 25 | * | * | * |
|  | Guadalupe | M | 16 | * | * |  |
|  | Julio | M | 21 | * | * | * |
|  | Maddie | F | 5 | * | * | * |
|  | Mercedes | M | 4 |  |  | * |
|  | Moheli | F | 24 | * | * | * |
|  | Paco | M | 14 | * | * | * |
|  | Pedro | M | 21 | * | * |  |
|  | Piedad | F | 22 |  |  | * |
|  | Sancho | M | 28 | * | * | * |
| *Lemur catta* | Berisades | M | 5 |  | * | * |
|  | Cap N'Lee | M | 8 |  | * | * |
|  | Cebes | M | 4 | * | * |  |
|  | Chandler | M | 11 | * | * |  |
|  | Chloris | F | 23 | * |  |  |
|  | Cleis | F | 24 |  | * | * |
|  | Cleonomis | F | 21 |  |  | * |
|  | Dorieus | F | 11 | * | * |  |
|  | Ginger | F | 2 |  |  | * |
|  | Herodotus | M | 5 | * | * |  |
|  | Ivy | M | 3 |  | * | * |
|  | Lilah | F | 4 |  |  | * |
|  | Nemo | M | 21 |  |  | * |
|  | Nicaea | F | 5 |  |  | * |
|  | Randy | M | 5 | * | * |  |
|  | Shasta | F | 3 | * | * |  |
|  | Sophia | F | 5 | * | * |  |
|  | Tellus | F | 4 |  |  | * |
|  | Teres | M | 16 | * |  | * |
|  | Tugger | M | 28 | * |  |  |
| *Propithecus coquereli* | Anastasia | F | 4 |  |  | * |
|  | Antonia | F | 10 |  |  | * |
|  | Conrad | M | 2 | * |  |  |
|  | Gaius | M | 4 | * |  |  |
|  | Gordian | M | 12 |  |  | * |
|  | Gratian | M | 6 |  |  | * |
|  | Irene | F | 4 | * |  |  |
|  | Jovian | M | 4 |  |  | * |
|  | Lucius | M | 6 | * |  | * |
|  | Marcus | M | 6 |  |  | * |
|  | Martinianus | M | 3 | * |  |  |
|  | Matilda | F | 3 | * |  | * |
|  | Pia | F | 9 |  |  | * |
|  | Romulus | M | 1 | * |  |  |
|  | Rupert | M | 2 | * |  |  |
|  | Rupilia | F | 12 | * |  |  |
|  | Tiberius | M | 21 |  |  | * |
|  | Wilhelmina | F | 2 | * |  |  |
| *Varecia variegata* | Alphard | M | 19 |  | * | * |
|  | Antlia | F | 22 | * | * | * |
|  | Aries | M | 4 | * | * | * |
|  | Avior | M | 3 | * | * |  |
|  | Borealis | M | 23 | * | * | * |
|  | Carina | F | 4 |  |  | * |
|  | Comet | M | 24 |  |  | * |
|  | Diphda | F | 19 |  |  | * |
|  | Esther | F | 2 | * | * |  |
|  | Grace | F | 12 |  |  | * |
|  | Hunter | M | 15 | * | * | * |
|  | Hydra | F | 3 | * | * |  |
|  | Little Dipper | F | 6 |  |  | * |
|  | Junior | M | 6 | * |  | * |
|  | Minias | M | 17 | * | * |  |
|  | Phoebe | F | 2 | * | * |  |

Figure S1. Schematic of the platform used in study 1 (from above). Subjects were centered at a stationing block (A) at the beginning of each trial. Subjects were then allowed to attempt to retrieve food from the choice locations (B). A vertical panel separated the choice locations.


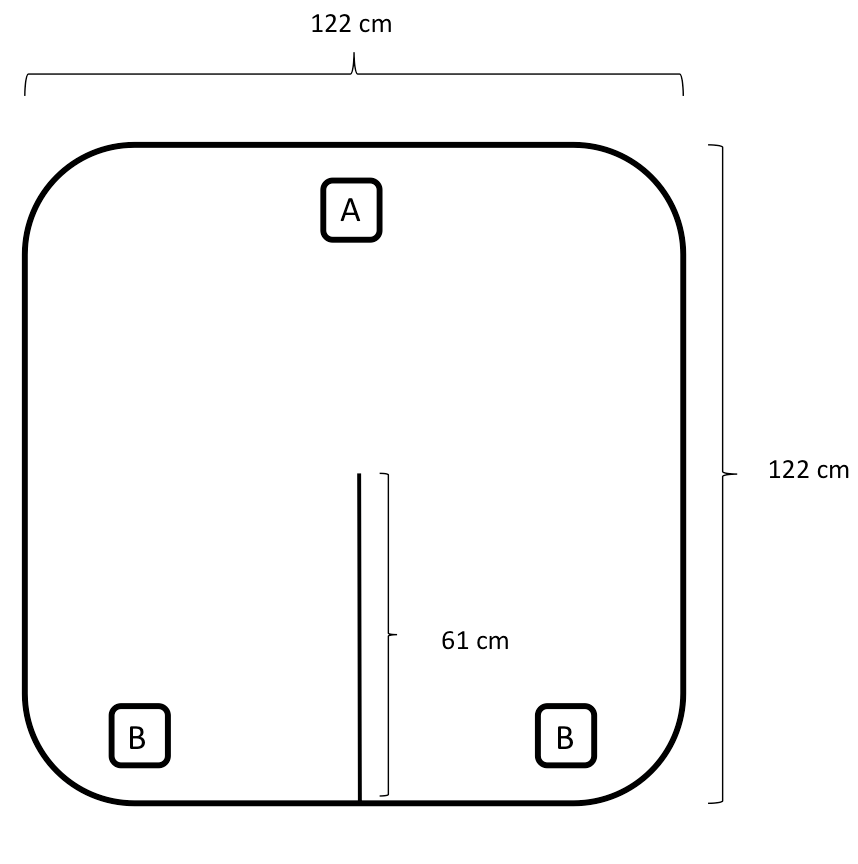

Supplement: File S1 — Supplemental analyses, Table S1, and Figure S1. (DOCX) [file pone.0066359.s001.docx]
